# Estimating malaria chemoprevention and vector control coverage using program and campaign data: A scoping review of current practices and opportunities

**DOI:** 10.7189/jogh.10.020413

**Published:** 2020-12

**Authors:** Johanna Nice, Honelgn Nahusenay, Erin Eckert, Thomas P Eisele, Ruth A Ashton

**Affiliations:** 1 MEASURE Evaluation, Centre for Applied Malaria Research and Evaluation, Tulane School of Public Health and Tropical Medicine, New Orleans, Louisiana, USA; 2 U.S. President's Malaria Initiative, United States Agency for International Development, Washington, D.C., USA; 3 RTI International, Washington, D.C., USA; 4 Centre for Applied Malaria Research and Evaluation, Tulane School of Public Health and Tropical Medicine, New Orleans, Louisiana, USA

## Abstract

**Background:**

Accurate estimation of intervention coverage is a vital component of malaria program monitoring and evaluation, both for process evaluation (how well program targets are achieved), and impact evaluation (whether intervention coverage had an impact on malaria burden). There is growing interest in maximizing the utility of program data to generate interim estimates of intervention coverage in the periods between large-scale cross-sectional surveys (the gold standard). As such, this study aimed to identify relevant concepts and themes that may guide future optimization of intervention coverage estimation using routinely collected data, or data collected during and following intervention campaigns, with a particular focus on strategies to define the denominator.

**Methods:**

We conducted a scoping review of current practices to estimate malaria intervention coverage for insecticide-treated nets (ITNs); indoor residual spray (IRS); intermittent preventive treatment in pregnancy (IPTp); mass drug administration (MDA); and seasonal malaria chemoprevention (SMC) interventions; case management was excluded. Multiple databases were searched for relevant articles published from January 1, 2015 to June 1, 2018. Additionally, we identified and included other guidance relevant to estimating population denominators, with a focus on innovative techniques.

**Results:**

While program data have the potential to provide intervention coverage data, there are still substantial challenges in selecting appropriate denominators. The review identified a lack of consistency in how coverage was defined and reported for each intervention type, with denominator estimation methods not clearly or consistently reported, and denominator estimates rarely triangulated with other data sources to present the feasible range of denominator values and consequently the range of likely coverage estimates.

**Conclusions:**

Though household survey-based estimates of intervention coverage remain the gold standard, efforts should be made to further standardize practices for generating interim measurements of intervention coverage from program data, and for estimating and reporting population denominators. This includes fully describing any projections or adjustments made to existing census or population data, exploring opportunities to validate available data by comparing with other sources, and explaining how the denominator has been restricted (or not) to reflect exclusion criteria.

Accurate estimation of intervention coverage is a vital component of malaria program monitoring and evaluation [1,2]. Information about intervention coverage enables national malaria programs (NMPs) to monitor progress towards coverage targets defined in national strategic plans, make implementation changes in response to low coverage, and to estimate the effectiveness of the malaria program. While large scale surveys such as Demographic and Health Surveys (DHS) or Malaria Indicator Surveys (MIS) are considered as the gold standard approach to generate estimates of intervention coverage [3,4], there is growing interest in maximizing the utility of program data to generate interim estimates of intervention coverage in the periods between successive MIS/DHS. However, despite the significant scale-up in malaria interventions since the 2000s, there is a lack of evidence to demonstrate best practices for estimating malaria intervention coverage among the population at risk using existing program data.

Throughout this article, we define program data as both the data collected by health providers on an ongoing basis (eg, antenatal care (ANC) visits, or health management information system (HMIS) data), as well as data collected during specific malaria intervention campaigns conducted by the NMP and implementing partners. During intervention campaigns, the number of individuals or households receiving the intervention is recorded, which can serve as the numerator in coverage estimates. However, estimating the denominator – which could be defined as the population eligible for the intervention, the population targeted, or the population at risk – presents additional complexities in settings where current and valid population data are not available.

Small-scale coverage surveys following intervention campaigns can also provide estimates of coverage. However, it may not be feasible to conduct coverage surveys in resource-constrained settings, and these surveys may not be adequately powered to generate population-representative estimates of coverage across different strata or risk groups. Furthermore, sampling frames used in post-campaign surveys still require some estimate of the underlying population at risk or the population targeted with the intervention, and therefore face some similar challenges in denominator estimation as program data.

This article presents a review of current practices and innovations to estimate malaria intervention coverage, specifically insecticide-treated nets (ITN), indoor residual spraying (IRS), intermittent preventive treatment in pregnancy (IPTp), mass drug administration (MDA), and seasonal malaria chemoprevention (SMC), using routinely collected data, or data collected during and following intervention campaigns. The study aimed to identify relevant concepts and themes that may guide future optimization of intervention coverage estimation using program data, with a particular focus on strategies to define the denominator. This review includes coverage estimates that are primarily for process evaluation and impact evaluation. For process evaluation, the focus is on understanding if the intervention is reaching the targeted population, where the denominator is the population eligible for the intervention of interest within a specific catchment area, and presumably, at risk for malaria. In contrast, the estimation of malaria program effectiveness requires data about overall population intervention coverage, where the denominator is the population at risk or the total population. Results are presented specific to each intervention type to allow an in-depth examination of coverage estimation practices. This includes an overview of the recommended coverage indicators followed by a quantitative and qualitative summary of the methodology employed by included studies. The discussion then summarizes key challenges across studies, as well as practical recommendations for improving current practices.

## METHODS

### Literature search method

A scoping review of current practices for measuring coverage of malaria interventions was conducted in 2018. It included English language literature published between January 1, 2015–June 1, 2018, identified through systematic searches of Pubmed, OvidSP (EMBASE & Global Health), and the Cochrane database of systematic reviews. Search results were reviewed to identify articles according to predefined criteria, primarily the inclusion of malaria intervention coverage estimates with a defined numerator and denominator. Secondary analyses, simulation studies, and reports of a solely qualitative nature were excluded. Malaria prevention and control interventions included ITN distribution, IRS, IPTp, MDA, and SMC. Malaria case management was excluded because coverage estimation relates to broader issues around population access to health services, the effectiveness of, access to, and quality of confirmatory diagnostic testing, and the effectiveness of surveillance systems to record malaria testing and treatment.

While data were extracted for all studies meeting the criteria above, only studies presenting intervention coverage estimates using program data or post-campaign surveys are included in the current article. Additional publications describing new developments in the use of programmatic data or denominator estimation in public health were identified from multiple sources, including PubMed searches, articles previously known to the author, and reference lists. Characteristics of all studies included in the scoping review are listed in Table S1 in the **Online Supplementary Document**, with a full description of the approaches to estimate coverage provided in Table S2 in the **Online Supplementary Document**. There is no formal assessment of study quality in line with current scoping review guidelines [5,6]; however, the advantages and disadvantages of different methodological approaches, in general, are discussed.

## RESULTS

### Selection of studies

**Figure 1:**
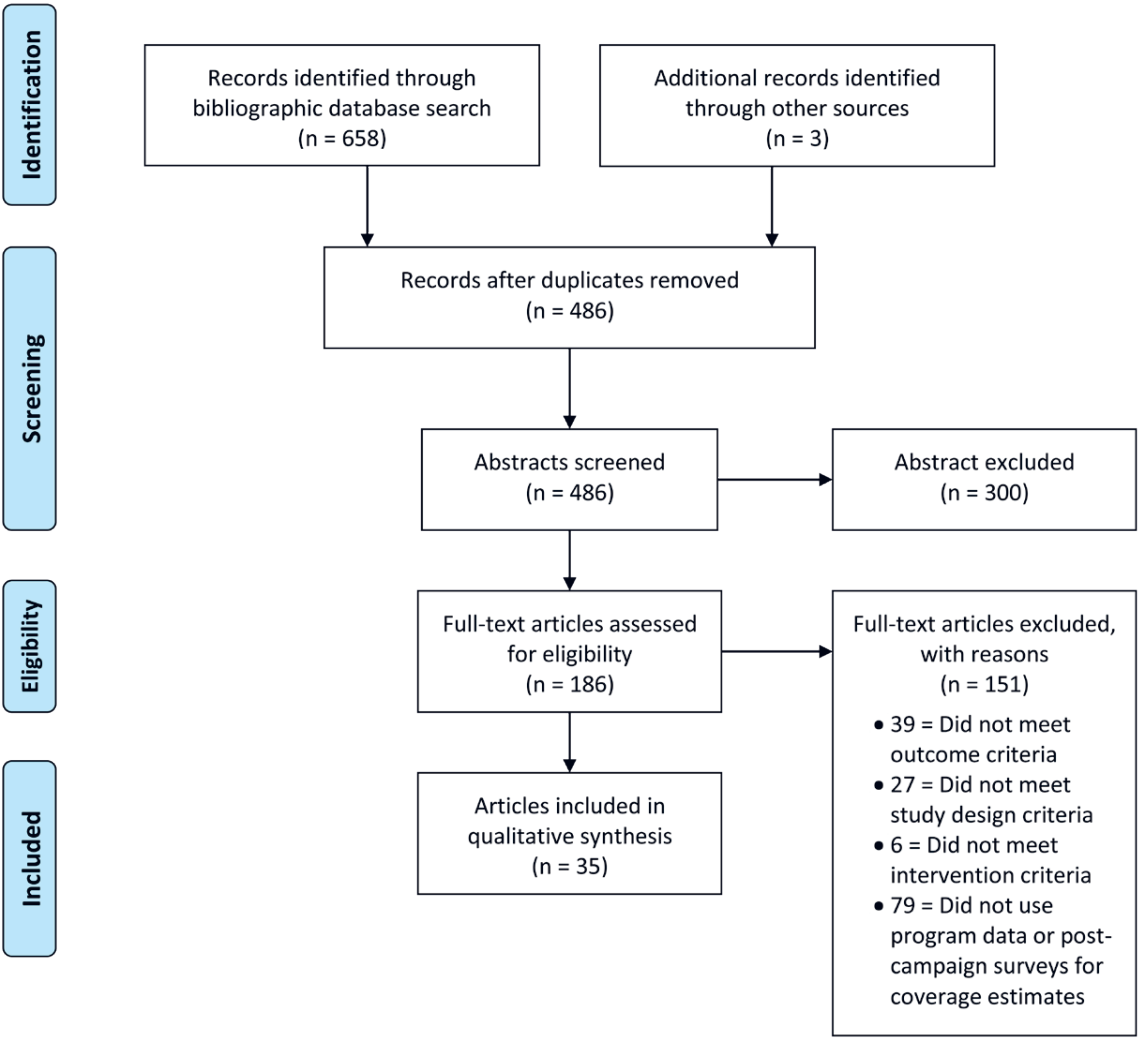
PRISMA flow diagram for studies assessing malaria intervention coverage 2015-2018.

A total of 658 articles were identified, 183 of which were potentially relevant after screening titles and abstracts. Two other studies that met the inclusion criteria were identified through the included studies, and one study that was not yet published was included. After full-text review, 35 articles met the inclusion criteria and 151 articles were excluded from the review. See **Figure 1** for the flow diagram for selected and excluded studies.

### Seasonal malaria chemoprevention

Seasonal malaria chemoprevention is designed to reduce the incidence of severe malaria in children living in areas with highly seasonal malaria transmission [7]. The World Health Organization (WHO) recommended indicator for monitoring SMC programs is the “proportion of children aged 3-59 months who received the full number of courses of SMC per transmission season”, using routine reporting system data [8]. While the recommended indicator assumes measurement of completed courses of SMC drugs in each cycle, the WHO's 2013 SMC field guide suggests instead defining coverage according to the first dose within each cycle [9]. The focus on recording the first SMC dose is a result of directly observed administration by the health care provider, with doses two and three given at home by the caregiver. An alternative approach to estimate SMC coverage is through post-campaign surveys, where a random selection of households are visited shortly after completion of the SMC campaign (ideally within one week) to measure reported participation in SMC, including completion of the full three day course [9].

Five studies reporting SMC coverage estimates, described in seven articles, were identified in the review [10-16]. Two studies included estimates derived from program data [12,14-16]. All five studies primarily relied on post-campaign surveys to assess SMC coverage, which used caregiver report of a child's participation in SMC as the numerator and the number of surveyed children eligible (based on age and residence) for the intervention as the denominator. All five studies additionally reported using programmatic tools, such as SMC cards, distributed to track participation of children in each round; however, data from these tools (retention of cards, coverage estimated from cards) was rarely reported [11-13].

Studies incompletely or inconsistently described how children meeting more specific ineligibility criteria, such as contraindications, were handled in the denominator. Two studies excluded children ineligible for SMC due to severe illness, known allergies to SMC drugs, or receipt of co-trimoxazole treatment, from the denominator [10,11]. Another study specified that children who were febrile on the day of the study were excluded from SMC and referred to clinics for malaria testing; however, the authors noted that community health workers did not consistently refer febrile children and withhold SMC [14]. The authors suggested an alternative definition of administrative coverage that would capture instances where febrile children were referred to facilities but found not to have malaria and consequently received SMC drugs at the facility, requiring modification of SMC cards to capture SMC given at clinics following negative malaria tests [14]. An additional challenge for denominators raised by authors regarded the potential exclusion of mobile populations from coverage surveys, such as those temporarily residing close to farmland for some or all of the SMC period [11-13].

Two studies triangulated results of coverage estimates from program data to estimates derived from post-campaign surveys [12,14-16]. One study in Burkina Faso used adjusted census data to estimate the eligible population for SMC; however, the adjustment method was not reported, and coverage regularly exceeded 100%; in contrast, the post-campaign surveys estimated coverage of 95% or lower depending on the SMC cycle [12]. In this context, it is difficult to determine if coverage exceeding 100% is a result of inaccurate denominator estimation or inclusion of children from outside the target geographical area or target age group in the numerator. Inclusion of children older than 59 months in post-campaign coverage surveys is suggested to estimate coverage among non-target children [11], and may also allow age heaping (whereby reported ages are rounded to attractive numbers) to be identified and addressed. Conversely, the second study, in Senegal, used Demographic Surveillance System (DSS) population estimates for the program data denominator and found that post-campaign surveys provided consistently higher estimates of coverage than program data. The authors hypothesized that denominator estimates from DSS data might have overestimated the target population, by including families who had temporarily migrated and were not present during the SMC campaign. However, DSS data are generally considered an up-to-date and reliable source of population denominators, though limited to the specific surveillance sites.

### Mass drug administration

Mass drug administration for malaria involves the administration of a full therapeutic course of anti-malarial medicine to a defined population within a specified time period and geographic region, regardless of the presence of symptoms or infection [17]. The WHO's 2018 Malaria surveillance, monitoring & evaluation reference manual does not include any MDA coverage indicators [8]; however, WHO technical and operational guidance is available for organizing a successful MDA campaign and suggests two methods to measure coverage [17]. 'Distribution coverage' is defined as the proportion of the targeted population who received the first dose of treatment in the specific MDA round and is calculated using MDA program data for each MDA round. The WHO suggests conducting a household census before the MDA campaign to generate a denominator of people in the target area. However, in settings where a pre-distribution census is not feasible, WHO recommends using a post-MDA coverage survey, which will generate both a numerator (number of surveyed individuals reported to have taken MDA drugs) and a denominator (number of surveyed individuals age-eligible for inclusion in MDA campaign) from a representative sample of the population [17]. Women in the first trimester of pregnancy are excluded from MDA campaigns, generating additional challenges in identifying and estimating the denominator.

The review identified ten studies with coverage estimates for MDA strategies targeting administrative areas or specific households [18-28]. The reviewed articles involved two to three rounds of two- or three-day courses of anti-malarials, and all but one study [24] involved direct observation of at least the first dose of treatment. Studies used program records [20,22,23,27], post-campaign surveys [26] or a combination of program records and post-campaign surveys [18,19,21,24,26,28] to estimate coverage. One study compared coverage estimates using capture-recapture methods to those from program and survey data [28].

Most studies included in the review provided estimates of programmatic coverage as the proportion of the target population who received MDA. However, coverage terminology was not used consistently, and denominator estimation methods were rarely described in detail. None of the included studies used the term 'distribution coverage' as defined by WHO [17]. Only two articles used the terms 'operational coverage' and 'effective coverage' [23,25], and only one defined the terms clearly [25]. One study used the term 'program coverage,' equivalent to 'operational coverage' [28]. It was common to include the full target population in the denominator, and separately note the percentage of non-participants who met exclusion criteria (eg, women in their first trimester of pregnancy, infants less than six months of age and individuals who are seriously ill or have known allergies to the MDA drugs) [18-20,22,24]. Two studies additionally tracked coverage among mobile populations, such as returning residents, migrants and visitors [20,22].

Studies relied on data collected at different points of the MDA campaign to estimate the denominator, including household enumeration conducted before [18,20,22-24] or during drug distribution [19,27], with the timing and completeness of enumeration affecting estimates. Despite using household registration data from a mass ITN campaign conducted approximately six months earlier, one study found that the targeted population was underestimated in urban areas, where the population is less stable [23]. Another study initially estimated MDA coverage needs using a denominator based on a census of the target population conducted by program staff, which was verified with local leaders in all operational districts [24]. This process of validation with local leaders yielded a markedly higher estimated population size than expected (184% increase). The authors suggest this may be due to overcrowding and multiple families sharing the same household. Despite the increase in the estimated population, the denominator was still underestimated due to the exclusion of several small minority communities that were not discovered until the second round of MDA. These communities were not initially identified as part of the enumeration area by the local leaders.

MDA in epidemic or complex emergency settings generally relies on existing population denominators, for example, from other recent health interventions [23,24]. In Sierra Leone, the target population was estimated from ITN mass campaign records from six months prior, with the numerator taken from program records (number of MDA courses distributed) to estimate coverage [23]. Estimates were triangulated with post-MDA campaign surveys conducted by an independent monitor [23]. In Liberia, where fixed-point distribution was used to give vouchers that could be exchanged for MDA drugs for two adults and five children, estimating coverage was challenging since neither the numerator (number of MDA treatments provided) nor denominator (target population at risk) could be readily estimated [24]. However, the authors used the program data to determine how many vouchers were distributed and how many were traded in for medication, by round, providing an MDA acceptance rate estimate.

### Intermittent preventive treatment in pregnancy

Intermittent preventive treatment in pregnancy with sulfadoxine-pyrimethamine (SP) is recommended in areas of moderate to high malaria transmission in sub-Saharan Africa. The WHO recommends using routine health information systems or household surveys [8] to monitor the proportion of pregnant women receiving each dose of SP, including the updated guidance regarding third and fourth doses [29]. When surveys are used to estimate IPTp coverage, numerators and denominators are defined from the surveyed population, but questions relating to IPTp are usually restricted to women who have had a live birth. When coverage is estimated using routine program data, the numerator for IPTp must come from ANC records at health facilities (including any pregnancies that did not result in live birth), and the denominator is likely to be estimated from census projections. The denominator for the number of women eligible for IPTp is also influenced by human immunodeficiency virus (HIV) infection rates since SP is contraindicated in women receiving co-trimoxazole prophylaxis [29]. As a consequence of these differences, IPTp coverage estimates from health facility records are most useful for process evaluation purposes, to understand if uptake of IPTp at ANC is adequate since the denominator is restricted to those women attending ANC. Assessments of IPTp impact generally require an understanding of IPTp coverage among the eligible population, incorporating both ANC access and IPTp uptake among ANC attendees.

In the review, 16 studies assessed coverage of IPTp interventions [30-46], but only two relied wholly on program monitoring data for coverage estimates. One study used program monitoring data from a pilot intervention of community- and facility-based delivery of IPTp [36], while another relied on ANC logbooks and monthly district reports [33]. Nine studies collected data on IPTp coverage using surveys, mostly facility-based, of which six studies confirmed respondent self-report via the use of routine data, including hospital records, logbooks, or ANC cards [30,35,37,39-41].

Coverage terminology was a challenge for IPTp studies identified in the review, with a range of indicators used to reflect the different number of rounds of IPTp that could be received. While most IPTp coverage estimates used ANC attendance as a denominator, only one study used the number of pregnant women attending their *first* ANC visit as the denominator, with the number of women receiving their first, and second, dose of IPTp as the numerators [33]. In general, the use of ANC attendance as a denominator results in an over-estimate of coverage if some women do not attend ANC at all or seek ANC services outside the public health sector. An additional indicator describing the number of missed opportunities for IPTp could be useful in settings where barriers to IPTp are at the provider-level, rather than as a result of low ANC attendance [42]. While IPTp with SP is not recommended for women who are taking co-trimoxazole prophylaxis, few studies reported whether these women had been excluded from the denominator used to estimate IPTp coverage [30,35]. Survey-based IPTp coverage estimates use women's recall of taking SP during an ANC visit; however, women may not be told the name of drugs prescribed during ANC visits, and consequently may not be aware if they receive IPTp [43]. Coverage indicators derived from both survey and program data should be sensitive to the local variations in service delivery across Africa, including the expansion of IPTp provision through community health workers [43].

Two studies used community and facility-based designs to estimate IPTp coverage. A trial of IPTp delivery methods in Nigeria used two different coverage estimation methods [36]. The numerator was derived from study-specific outcome forms completed by community and facility-based staff to capture SP doses, then aggregated by facility within District Health Information Software 2 (DHIS2). A census of the intervention area was conducted for the intervention arm denominator, including the number of eligible and ineligible pregnant women, though operational issues prevented complete enumeration of all intervention areas. In contrast, control arm areas estimated the denominator using official population estimates (Sokoto State Government), assuming 5% of the population was pregnant, and that 94% of pregnant women were eligible for the intervention, prorated over the eight months of the project. The authors noted discrepancies between official census and intervention area enumeration estimates that suggested the official census data may under-estimate the number of eligible women. A second study, which identified eligible survey respondents from a health and demographic surveillance site, suggested that IPTp coverage may have been overestimated due to underrepresentation of the rural population in the study district, and that rural women are less likely to receive IPTp [30].

### Insecticide-treated net

Ensuring universal vector control coverage for all people at risk of malaria is a pillar of the Global Technical Strategy (GTS) for malaria; and includes 100% access to, and use of, either IRS or ITNs by populations at risk of malaria [47]. The GTS minimal recommended indicator for vector control intervention outcomes is the proportion of the population at risk of malaria sleeping under an ITN or living in a house sprayed by IRS in the previous 12 months [8]. This indicator can be measured independently with a household survey, but is preferably assessed in combination with routine monitoring data [48]. Other indicators recommended to be sourced from routine monitoring data include the proportion of the population at risk potentially covered by distributed ITNs; and the proportion of targeted risk group receiving ITNs.

Household surveys continue to be the most common method for estimating ITN coverage, and there are a range of recommended ITN ownership and use indicators [4]. However, recent evidence suggests that population access to ITNs–where the denominator is people, not households–is a better indicator of universal coverage than the proportion of households owning at least one ITN for every two people [49].

The scoping review identified eighty studies with defined coverage estimates for ITN interventions, mostly derived from cross-sectional household surveys. A few studies used surveys administered through other venues, including health facilities [50], plantations [51], and schools [52]. Only eight studies relied on program monitoring or routine surveillance data [33,53-59].

Most studies using program data reported on the proportion of the population potentially protected by ITNs using NMP reports on net distribution to calculate the numerator and population projections for the denominator [53,56,58,59], though few studies explicitly described the assumptions used for estimating either the numerator or denominator. Among those that provided full details, one study in Ghana calculated the proportion of the population (all ages) potentially protected by long lasting insecticidal nets (LLIN) each year from 2005 to 2015 using district records and assuming that each LLIN distributed covered 1.8 persons and lasted three years. The denominator was calculated using the 2010 Ghanaian census and the United Nations growth rate for Ghana [53].

One study in Kenya conducted school-based post-campaign surveys in order to quickly obtain ITN ownership and use coverage estimates, with the limitation that responses could not be verified by observation [52]. Lot quality assurance sampling (LQAS) was employed by a handful of post-campaign surveys [60-63]. A small study validating a rapid assessment tool for malaria prevention used LQAS to identify areas that were not reaching targets of intervention coverage and use [61]. Another applied LQAS methods to a secondary analysis of a nationally representative household survey data set to estimate community-level coverage by considering each cluster as a lot, and assigning a pass-fail threshold for different ITN coverage targets, using a 20%-point margin between target and minimally acceptable result [63]. LQAS was also used to determine whether net ownership and use thresholds were met after a national ITN distribution campaign in Mozambique, with findings similar to estimates generated by the NetCalc tool [60]. NetCalc uses programmatic data describing the number of nets distributed, estimated durability of nets, pre-distribution net coverage, and estimates of population and household size to generate predictions of expected net coverage.

Alternative approaches to generate rapid and low-cost malaria intervention coverage estimates include surveys among easy to access groups (EAGs) and mobile phone surveys. EAGs are defined as representative subsets of the population or at-risk groups that assemble at easily accessible locations such as schools or health facilities, or during public health intervention activities such as catch-up vaccination campaigns [64]. The utility of EAGs for monitoring and evaluation has not been fully explored, though a review found estimates from EAGs commonly over-estimated population coverage values, but with varying degrees of accuracy [64]. Concerns relating to the representativeness of EAG samples could be alleviated by inclusion of a small calibration survey, to generate a correction value that can be applied to EAG sites. Additional hybrid sampling approaches and strategies to minimize bias from the use of EAG data are presented by Sesay et al. [64]. Mobile phone surveys offer another survey approach that is faster and lower cost than household-based surveys. A pilot of phone-based surveys in Tanzania to assess ITN coverage suggests that by use of non-response adjustments such as raking or post-stratification, representative estimates of ITN coverage can be achieved (Worges et al, in preparation).

### Indoor residual spraying

The leading coverage indicator reported by IRS programs is defined as the proportion of targeted or found households or structures which were successfully sprayed. This a clear example of a coverage indicator that may be useful for monitoring program implementation but cannot be easily extrapolated to understand IRS among the population at risk and consequently to evaluate the impact of IRS. Both the numerator and denominator for this indicator are generated from spray operations reporting [65]. Additional strategies described by the WHO IRS operational manual include conducting baseline geographic reconnaissance and census to identify households in the targeted areas, and use of house spray cards to track participation over multiple rounds [65]. The manual also recommends a separate estimate of IRS coverage through post-campaign surveys, where both the numerator and denominator are generated by the survey [48].

Eighteen studies reporting IRS coverage were identified in the review [27,58,59,66-80]. Coverage estimates were more commonly obtained through post-campaign household surveys [27,66,69-71,74,76,79,80] than routine IRS program records [58,59,68,72,73,77] or study records [75]. Two studies reported coverage from both household surveys and program records [67,78]. Three studies piloted innovative methods for improving IRS implementation, including mobile phone-based household sensitization techniques [73], the use of satellite enumeration and spatial aids to assist spray teams [66], and integrating IRS within a community-based rural health services program [77]. One study assessed the impact of multiple targeted interventions, including IRS, in transmission foci [27].

The most commonly reported indicator using program data was the proportion of enumerated structures sprayed (per spray cycle) using spray campaign data [59,67,68,72,73,77,78]. None of the reviewed studies reported the proportion of population at risk sleeping under an ITN or living in a house sprayed by IRS in the previous 12 months, potentially due to the difficulties in translating program data to household-level ownership measures [81]. Two studies reported estimates for the proportion of the population at risk protected by IRS, using routine monitoring data [59,77].

Few of these studies discussed the representativeness of estimates or inclusion of migrant and mobile populations. Survey response rates were rarely presented or discussed, although one study reported that almost one-third of households were not available to interview, and of those reached, only 62%-80% were willing to be interviewed [66]. Nwe et al. (2017) reported IRS coverage in Myanmar based on NMP district estimates for 2010-2014 and discussed the difficulties of accessing relevant data on migrant and mobile populations [58]. Another study noted that households were unable to be sprayed due to residents working in their fields during the spray campaign, resulting in particularly low coverage [73].

Two studies used multiple data sources to triangulate coverage estimates obtained during routine program implementation. A study in Uganda triangulated data from cross-sectional household surveys, routine cohort assessments, and monitoring data from the NMP, finding high IRS coverage by administrative and cohort data, but lower coverage from household survey data [78]. However, the household survey was conducted concurrently with IRS implementation, so surveyed households may not have yet been reached by IRS spray teams, emphasizing the importance of timeliness for drawing estimates. Citing concerns that administrative coverage estimates may underestimate the actual coverage, a study in Namibia compared IRS coverage reported using routine NMP data against household survey data [67]. However, the estimates used were not directly comparable as the program data considered structures as the sprayed unit, while the survey considered households as the sprayed unit.

IRS is often targeted based on administrative areas or may even be targeted according to environmental or ecological zones, the boundaries of which may not be apparent to spray teams in the field. A field-based enumeration approach has traditionally been used to guide indoor residual spray operations, which is often completed without validation that the entire targeted area was indeed enumerated [82]. Bridges et al. (2018) assessed the accuracy of satellite-based enumeration to identify sprayable structures in Zambia [66]. The sensitivity of satellite enumeration was assessed by dividing the total houses enumerated from satellite imagery by the total houses found during field-based enumeration. The study found that satellite enumeration underestimated the number of structures in the sampled areas. However, with an overall sensitivity of 94%, the authors concluded that satellite enumeration is an accurate and more cost-effective and scalable alternative to field-based enumeration methods for planning and monitoring IRS campaigns, with potential application to other interventions (ITNs, MDA, vaccination campaigns).

### Innovations in estimation of population denominators

Increased availability of satellite-derived imagery, use of global-positioning technology in field activities, and developments in statistical methodology and computing power have contributed to innovations in estimation of population denominators for settings where reliable and contemporary population census data are not available [83].

Top-down statistical methods allow census data to be disaggregated to a higher resolution through approaches such as dasymetric mapping [83,84], and small area estimation [85]. Model-based approaches to re-weight census data across census units have been used to generate high-resolution maps of estimated population distribution, with resulting gridded population data freely available from WorldPop [86-88]. Data from large-scale surveys and other sources have also been used by the Malaria Atlas Project (MAP) to generate high-resolution predicted surfaces for indicators such as accessibility, household construction, and relative vector abundance [89-91], which can be useful in defining the population in need of various malaria interventions. However, the reliability of top-down estimation methods is dependent on the availability of recent and high-quality input data and may not be appropriate in areas with high population mobility.

Bottom-up methods describe approaches using high-resolution satellite data to define settlement patterns or individual households. Both manual and automated methods to enumerate households have been piloted [66,82,92,93]. These methods require obtaining high or very-high resolution satellite imagery and estimates of mean household occupancy in order to generate population estimates [94]. The use of satellite imagery to estimate denominators has particular utility for IRS campaigns, but is limited in utility in urban settings with multi-level buildings or complex roof patterns, or where houses are obscured by tree canopy or cloud cover in satellite imagery. Bottom-up population estimates have been recommend to complement census and other enumeration activities, emphasizing that methods used to generate estimates should be transparent, and the importance of engaging relevant stakeholders to minimize political sensitivities related to population estimates [83].

Capture-recapture methods, initially developed by ecologists to estimate population sizes, have potential utility in estimating denominators where repeat samples of the same population are available. Finn et al. used a capture-recapture approach to estimate the total number of households that should have been visited by intervention teams during MDA activities in Zambia [28]. The authors matched two independent lists of people, one list from the MDA program data and a second list from a cross-sectional survey conducted in the intervention area, and estimated the total households in the targeted area using the *Schnabel* estimation method [28]. Using the estimated total households as the denominator yielded similar epidemiologic and household coverage estimates to the household post-campaign survey and a satellite enumeration method, but all were lower than estimates derived solely from MDA program data. Where individual-level data are captured in registers during successive rounds of intervention delivery, these capture-recapture estimation techniques offer another alternative to estimating the true denominator population.

Researchers have begun to produce dynamic estimates of population denominators, incorporating information about internal and international migration patterns, or seasonal movements of populations for agricultural and other reasons [95,96]. The use of anonymous and aggregated mobile data can assist in describing population distribution and movement [96,97]. Seasonally-appropriate denominators may be particularly useful for planning and estimating coverage of interventions such as SMC, vaccination campaigns and net distributions in settings with temporally-dynamic populations.

Gravity models have potential utility in estimating health facility catchment populations by incorporating information about estimated population distribution, distance to facilities, and facilities' relative attractiveness [98,99]. Modified gravity models have been used to estimate facility catchment populations in Haiti, Botswana, and Mozambique (E. Cameron, personal communication).

## DISCUSSION

This scoping review aimed to provide a situational analysis of current practices for assessing the coverage of major malaria prevention and control interventions in endemic settings, with a focus on the use of program data and small-scale post-campaign surveys. While cross-sectional surveys such as DHS and MIS remain the gold standard for population-representative estimates of intervention coverage, there is potential to complement these periodic estimates with routinely collected data (eg, IPTp) or data collected during intervention campaigns (eg, SMC, MDA, IRS, ITN distribution). In particular, program data can provide useful information about acceptance rates among targeted individuals (using campaign data to define the proportion of people reached who participated). However, true coverage estimates require an estimate of the population at risk (often itself based on proportions of the estimated population) and the proportion eligible for the intervention. Various options for denominators examined in this review include the use of census projections, validation of census estimates by local leaders, use of household census data collected by other health programs or intervention activities, and use of satellite-based household enumeration.

The review identified a lack of consistency in how coverage was defined and reported for each intervention type, with denominator estimation methods not clearly or consistently reported, and denominator estimates rarely triangulated with other data sources to present the feasible range of denominator values and consequently the range of likely coverage estimates. A summary of key challenges identified by the review in generating numerator and denominator values for coverage estimates, as well as potential solutions, are provided in **Table 1**. Not all the solutions proposed in **Table 1** may be feasible or appropriate for all settings, especially in large scale implementation; however, the overarching recommendations for coverage estimation provided in this discussion can be incorporated with minimal effort.

**Table 1:** Challenges in coverage estimation using program data and/or post-campaign surveys

	Seasonal Malaria Chemoprevention (SMC)	Mass Drug Administration (MDA)	Intermittent preventive treatment in pregnancy (IPTp)	Indoor Residual Spray (IRS)
**Numerator challenges**	• SMC coverage using program records only includes ingestion of the day 1 dose of SP+AQ, not completion of the full 3-d course of chemoprevention.	• MDA coverage often uses only data relating to ingestion of the day 1 dose, not completion of the full course of MDA. • Estimating coverage is particularly difficult if drugs are provided at distribution point to household representatives, rather than to individual household members.	• Survey-based estimates may underestimate IPTp coverage by limiting to receipt of SP from 'skilled providers' only, and are subject to recall bias. • Routine monitoring data may not capture the updated WHO recommendation that women receive at least three rounds of IPTp.	• Poorly demarcated target boundaries affect IRS implementation and coverage estimates. • The relevant time period for recall of IRS varies according to the insecticide used.
**Potential numerator solutions**	• If SMC cards are used, retention of these cards should be reported in coverage surveys, as well as coverage according to data on these cards.	• Specify coverage estimates relate to day 1 dose only. • Amend MDA strategy to DOT on all doses, resources permitting. • Check drug blister packets during post-campaign surveys to estimate proportion ingesting all doses.	• Electronic data systems at ANC clinic could facilitate linkage of data from each ANC visit.	• Spatial aids may assist in accurate identification of spray-targeted areas on the ground. • Surveys including IRS recall questions should be cognizant of insecticide used and effective period.
**Denominator challenges**	• Denominator may change from start to end of SMC period, some families may not remain resident for the whole period. • Off-target distribution of SMC is common.	• Census denominators may not be up to date, particularly in urban areas which tend to have higher growth rates. • Population denominators do not always state whether they include ineligible individuals (eg, pregnant women). • While local leaders can assist in validating population estimates, this may not be effective for incorporation of minority or mobile populations.	• Wide range of denominators presented in the literature when estimating IPTp coverage. • Population denominators often do not state whether they include ineligible individuals (eg, receipt of co-trimoxazole).	• Program coverage denominator is often “number of structures found by spray team,” which may not capture all target structures. • Migrant and mobile populations may be missing from denominators.
**Potential denominator solutions**	• Any cross-sectional post-campaign surveys should include children beyond the target age range, to estimate intervention coverage among older children.	• Triangulating denominator estimates from multiple sources, or validating by micro-census can assist in generating feasible ranges for the denominator.	• Use of ANC visit 1 as a denominator could aid understanding of IPTp uptake among ANC attendees.	• Clear definition of either household or structure as denominator. • Satellite imagery can assist in enumeration of target areas.

ANC – antenatal care; DOT – directly observed treatment; IPTp – intermittent preventive treatment in pregnancy; IRS – indoor residual spray; MDA – mass drug administration; SMC – seasonal malaria chemoprevention; SP – sulfadoxine-pyrimethamine; SP+AQ – sulfadoxine-pyrimethamine and amodiaquine; WHO – World Health Organization

For drug-based interventions, there are additional challenges in defining the numerator for coverage estimates, particularly if estimates only include the first dose of each round. While there are logistical challenges in collecting information on each dose in program records (directly observed treatment (DOT) is generally limited to the day-one dose), post-campaign surveys are better suited than program data to measure adherence to the full SMC or MDA course. Post-campaign coverage surveys are generally smaller-scale and more focused than DHS and MIS, aiming to measure intervention coverage and uptake specifically, and with fewer problems related to recall bias than DHS/MIS.

Limitations of this review include the focus on articles published in peer-reviewed journals, which may have excluded relevant grey literature describing activities conducted by NMP or implementing partners as part of routine monitoring and evaluation, rather than as specific research activities. Additionally, while malaria case management is a core intervention in all NMPs, it was beyond the scope of the current review.

The first main recommendation from this review relates to fully describing any projections or adjustments made to existing census data and exploring opportunities to ground-truth available data by comparing with other sources. For denominators based only on census projections, the year of the census, annual growth rate, and any other adjustments or assumptions (eg, proportion currently pregnant) should be clearly reported. Furthermore, internal migration may make use of census projections within individual districts and health facilities unreliable [100]. A range of methods can be used to ground-truth census projections, such as the use of microcensus [92], collaborating with local leaders to validate community population estimates [24], or using population estimates generated by other programs (eg, vaccination, ITN distribution) [23,101]. The NMP in Ghana has used population data collected electronically during registration for an ITN campaign to inform SMC denominator estimates in microplanning activities [102]. Obtaining population numbers in collaboration with local leaders may improve population estimates. However, care should be taken to ensure that minority groups that may not be represented by community leaders are also included in the count [24], and implementers should be cognizant of potential sensitivities and biases using this approach. A pilot of denominator estimation in collaboration with local leaders in Jigawa State, Nigeria will provide further information about the utility of this approach to generate reliable denominators and improve coverage estimates from SMC program data (Arantxa Roca, personal communication). High coverage is essential for MDA in elimination settings to minimize development of anti-malarial drug resistance. As a consequence, more intensive validation of census data may be required before MDA in elimination areas, including estimation of migration in and out of the MDA target area, or even conducting a pre-campaign census [103].

The second main recommendation relates to defining eligible and ineligible individuals and explaining how the denominator has been restricted (or not) to reflect these criteria. Since the target population for SMC is children ages 3–59 months, estimation of the denominator is challenging in settings without census data on this specific age group, or where parents/caregivers may not know children's exact ages. If post-campaign coverage surveys are planned to estimate SMC coverage, the inclusion of children older than 59 months in the survey is suggested to estimate coverage among non-targeted children [11]. Estimating the denominator for IPTp faces similar challenges; however, an alternative approach is to use a combination of United Nations (UN) population estimates and predictive malaria endemicity maps to estimate the number of malaria-exposed births in a defined geographical unit [104]. For chemoprevention intervention denominators, in particular, it is important to specify the inclusion or exclusion of individuals who are ineligible for the intervention due to contraindications. While likely a minor contributor to inconsistent coverage estimates, incomplete reporting of calculation methods limits conclusions and comparisons between studies [105]. When it is not possible to conduct a full census before implementation, the most feasible approach is to include the full age-eligible population in the denominator, and separately note the percentage estimated to be ineligible due to specific exclusion criteria (often obtained at the time of drug distribution). In areas with generalized HIV epidemics, ineligibility for IPTp due to co-trimoxazole prophylaxis (which is recommended rather than IPTp for pregnant women living with HIV in Africa to prevent malaria complications in infants [106]) may have a more substantial impact on the denominator. A potential solution is to include women taking co-trimoxazole prophylaxis in the IPTp denominator but report the proportion of all pregnant women that they represent. Alternatively, if these women are excluded from the denominator, a separate indicator could be reported to describe the proportion of pregnant women receiving co-trimoxazole prophylaxis.

Where interventions are targeted to households rather than individuals, a different set of challenges and potential solutions are identified. IRS coverage has commonly been defined as the proportion of found eligible structures that were sprayed. However, using “found structures” as the denominator risks overestimating coverage and erroneously excluding some geographical areas. This is further complicated by inconsistencies in the use of household or structure as the denominator. The IRS taskforce of the RBM Monitoring and Evaluation Reference Group is compiling guidance on denominator selection for the purpose of IRS coverage estimation, expected in 2020. Enumeration of households or structures using satellite imagery offers a more rigorous approach to defining the denominator than simply using those found by spray teams, and is less costly than a comprehensive field-based enumeration of household prior to IRS [66,82]. However, the use of satellite data for enumeration may be challenging in urban and forested areas due to difficulties identifying individual households from imagery.

## CONCLUSIONS

This scoping review demonstrates that while program data have the potential to provide intervention coverage data to complement estimates generated by periodic large-scale cross-sectional surveys, there are still substantial challenges in selecting appropriate denominators. To improve reporting and comparability of malaria intervention coverage, it is recommended to provide clear definitions of the numerator and denominator used, state the data sources, and any estimation methods or projections applied, as well as relevant inclusion and exclusion criteria. Regardless of the intervention being assessed or primary data source used, efforts should be made to triangulate coverage estimates using multiple data sources [100], and household surveys remain the gold standard method to estimate coverage of ITNs and IPTp. While there have been promising developments in modeling methods and the creation of different spatial population data sets, inconsistencies between data sets remain, particularly in low-income settings [107]. Alternative strategies such as the use of ANC attendees as sentinel populations [108,109], or easy access groups surveys [64] could also provide interim estimates of intervention coverage to complement DHS and MIS survey estimates.

## Additional material

Online Supplementary Document
