# Supplementary material for: Estimating malaria chemoprevention and vector control coverage using program and campaign data: A scoping review of current practices and opportunities
Source: J Glob Health. 2020 Jul 28;10(2):020413. doi: 10.7189/jogh.10.020413 (PMC7568932; doi:10.7189/jogh.10.020413)
Supplement: Online Supplementary Document [file jogh-10-020413-s001.pdf]

Online Supplementary Document

Nice et al. Estimating malaria chemoprevention and vector control coverage using program and campaign data: a scoping review of current practices and opportunities

Table S1. Characteristics of studies reporting coverage estimates using program data

Study	Study Setting	Study Context	Intervention Details	Study Purpose	Study Design	Sampling Methods	Sample	Coverage data source	Indicators
Seasonal Malaria Chemoprevention									
Compaore 2017	Burkina Faso<br><br>Subnational: Highly seasonal malaria transmission	Routine Implementation	4 SMC rounds with SP+AQ at monthly intervals to children aged between 3 and 59 months	To assess implementation fidelity of 2014 & 2015 SMC campaigns (extent to which program adhered to implementation plan, coverage of activities & targeted children)	Mixed methods study with caregiver-reported SMC coverage assessed via cross-sectional survey six months after the last SMC round, as well as administrative data	Simple random sampling using the HDSS database listing of SMC age-eligible (2-62 months at time of SMC campaign) children in the health district.	284 caregivers (284 children) in Kaya health district	Administrative data; Household Survey + *SMC administration cards*	• Proportion of children who received the first dose of each treatment round during the transmission season, calculated per treatment round<br>• Proportion of children who received four complete courses during the transmission season.
Ba 2018; Cisse 2016; Ndiaye 2016;	Senegal<br><br>Subnational	Impact Evaluation	3 SMC rounds with SP+AQ at monthly intervals to children aged between 3 and 120 months	To determine the effectiveness of SMC in Senegalese children up to ten years of age (2008-2010 cycles); and to inform ongoing implementation	Stepped Wedge Cluster RCT with caregiver reported SMC coverage assessed annually via cross sectional surveys at the end of the transmission season, as well as through administrative data. Analysis accounted for survey design	Census of target population (DSS) + Two stage cluster sampling (methods varied each year, but generally, health posts were selected with PPS or SRS and households within each post were selected from DSS database by SRS)	1,019 children (2008); 3,397 children (2009); 882 children (2010) in 54 health posts.	Demographic surveillance system; Administrative data; and Household surveys + *SMC administration cards*	• Proportion of children who received three monthly SMC treatments each year among those surveyed
Mass drug administration in areas approaching elimination or areas with accelerated malaria elimination efforts due to the threat of multidrug resistance									
Adhikari et al 2017	Lao PDR	Pilot: Targeted Malaria	3 rounds of MDA with 3day course	To explore the factors associated with participation in MDA as part of	Clinical trial with cross-sectional questionnaire conducted one	2016 Census of all households in targeted villages. One adult from each	973 out of 1017 villagers eligible for participation	MDA campaign records; Post-MDA	• Proportion of respondents who participated in all three rounds of MDA and blood survey out of all eligible respondents

Study	Study Setting	Study Context	Intervention Details	Study Purpose	Study Design	Sampling Methods	Sample	Coverage data source	Indicators
	Subnational: 2 TME intervention villages in Savannakhet Province close to the Vietnam border	Elimination (TME)	of DHAP[1] and PQ_{LD}[2] given as DOT at monthly intervals to entire eligible population[3]	targeted malaria elimination (to interrupt local *P. falciparum* transmission).	month after the third round of MDA to understand reasons for participation (July and August 2016). Baseline and quarterly blood surveys to measure parasitemia.	household selected for survey.	in MDA. 158 adults from all 158 households in the 2 intervention villages	Househol d survey	• Proportion of respondents who participated in at least one round of MDA and blood survey
Ali et al 2017	Zanzibar<br><br>Subnational: 4 shehias (smallest administrati on units) identified as high transmission hotspots in Pemba and Unguja islands, Zanzibar	Pilot Study	1 round of MDA with 3 day course of DHAP, AP[4] or artesunate–amodiaquine (depending on drug availability and age) given as DOT on first day to entire eligible population[5]	To measure programmatic coverage and compliance of MDA using artemisinin-based combination therapy (ACT) in areas identified as hotspots through Zanzibar's malaria case notification surveillance system	Feasibility study with cross-sectional questionnaire conducted two weeks after MDA distribution.	Systematic random sample of eligible households ordered by sub-shehia from a list for each shehia *(list source not described – unless it's the enumeration records from MDA campaign?*).	9076 participants in 2001 households enumerated for MDA; 2009 participants in 413 households enumerated for post MDA survey	MDA campaign records; Post-MDA household survey	• Proportion of targeted population that received treatment out of all people enumerated in four districts during MDA;<br>• Proportion that reported completing all three doses of treatment out of people surveyed post MDA.
Deng et al 2018	Union of Comoros<br><br>Subnational: Anjouan Island. Strong regional and seasonal transmission variations	Impact Evaluation	3 rounds of MDA with 2 day course of AP, with or without PQ_{LD}, given as DOT at monthly intervals to entire	To investigate outcomes of MDA of AP with or without PQ (primaquine)	Observational study comparing outcomes between treatment cohorts on Anjouan island (AP vs AP+PQ_{LD}) pre and post MDA (Oct-Dec 2012).	Census of Anjouan island households *(conducted by malaria control team)*	97,164 people in districts receiving AP+ PQ_{LD} MDA; 224,471 in districts receiving AP MDA; 81,212 travelers at	MDA campaign records	• Proportion of population that received each round of MDA out of total population<br>• Proportion of travelers that received therapeutic dose of AP out of all travelers

[1] Dihydroartemisinin piperaquine
[2] Single low dose primaquine
[3] Excluded infants under 6 months, pregnant women, and severely ill people
[4] Artemisinin–piperaquine
[5] Excluded infants under 2 months, and pregnant women in the first trimester of pregnancy

Study	Study Setting	Study Context	Intervention Details	Study Purpose	Study Design	Sampling Methods	Sample	Coverage data source	Indicators
			eligible population[6]				the airport and wharfs		
Kajeechiwa et al 2016	Myanmar<br><br>Subnational: Four Karen villages located within 10 km of the Thailand Myanmar border; High relative parasitemia rates	Pilot: Targeted Malaria Elimination (TME)	3 rounds of MDA with 3 day course of DHAP plus PQ_{LD} given as DOT at monthly intervals to entire eligible population[7]	To identify factors associated with acceptance of the complete drug administration (three rounds of three doses of antimalarials), part of the drug administration or no participation at all.	Acceptability study with cross-sectional questionnaire conducted one to six months after MDA distribution (2013, 2014).	Household heads or their representatives were selected in each household in each study village; *(No mention of how households were enumerated or if all households were able to be interviewed*.)	388 respondents	MDA campaign records; Post-MDA household survey	• Proportion of respondents that completed three consecutive doses of the antimalarials required for the complete clearance of parasites in any one round out of total respondents.<br>• Completed three rounds of three drug doses each; did not participate at all; and participated but did not complete the full course of 9 doses
Tripura et al 2018	Cambodia<br><br>Subnational: Four malaria-endemic villages in western Cambodia	Pilot Study	3 rounds of 3 daily doses of DHAP given as DOT at monthly intervals to entire eligible population[8]	To evaluate the effectiveness and safety of 3 rounds of mass drug administration using DHAP to interrupt malaria transmission	Cluster randomized controlled trial comparing outcomes between intervention and control villages	Census of study village population	2268 eligible respondents	MDA campaign records	• Proportion of study participants with at least one complete round (two or more rounds; or all three rounds) of MDA treatment out of eligible study participants<br>• Post MDA treatment of returning residents, migrants or visitors
Mass drug administration in epidemic or complex emergency settings									
Aregawi et al 2016	Sierra Leone<br><br>Subnational: 24 chiefdoms (sub-districts) in six rural districts; and	Pilot Study	2 rounds of MDA with ASAQ[9] in districts affected by Ebola during December 2014–January	To evaluate the impact of MDA on malaria morbidity and burden of cases presenting as Ebola suspected patients in the sub-districts targeted for MDA	Observational study comparing trends of malaria cases and Ebola alerts during the post-MDA weeks compared to the pre-MDA weeks in MDA-	Random sample of one eligible health center per chiefdom.<br><br>Eligible health centers were those that were functional, with diagnostic services. Health	2,899,500-3,043,168	MDA Campaign records (*National Malaria Control Program records*)	• Proportion of full ASAQ treatment courses distributed among targeted population.<br>• Proportion of people who took the first dose of the medicine under DOT on the day of visit by the drug distributors among the total number of people

[6] Excluded infants under 6 months, women in first trimester of pregnancy and people with liver or kidney diseases
[7] Excluded infants under 6 months and women in first trimester of pregnancy
[8] Excluding infants under 6 months, pregnant or lactating women, and anyone with acute health problems
[9] Artesunate/amodiaquine

Study	Study Setting	Study Context	Intervention Details	Study Purpose	Study Design	Sampling Methods	Sample	Coverage data source	Indicators
	30 zones in the Western Area, Freetown (10 zones in the Rural and 20 in Urban areas)<br>High malaria transmission		2015, given as DOT on first day to entire eligible population[10]		and non-MDA-chiefdoms	centers with incomplete data were replaced by other health centers from the same chiefdom.			who received full treatment courses.
Kuehne et al 2016	Liberia<br>Subnational: Four administrative zones of Monrovia with high Ebola incidence	Pilot Study	2 rounds of MDA with 3 day course of ASAQ given at fixed points of distribution at monthly intervals to population[11]	To describe the scale of the MDA; to evaluate community acceptance of the strategy and adherence to treatment; and estimate the effectiveness of the intervention with regards to the reduction of fever cases	Cross-sectional study with phone survey conducted one week after each round of MDA distribution.	One in every 200 households that was randomly selected for telephone follow-up through systematic sampling during Round 1 of MDA voucher distribution	Of the 365 selected households, 222 (61%) were reached by phone in both rounds. (1236 household members living there at both rounds; 1643 in one or both rounds)	MDA campaign records; structured questionnaire	• Household members who received sufficient ASAQ-CP during round 1 or round 2 of MDA<br>• Household members who reported starting treatment and household members who adhered to the full course of treatment
Mass drug administration in other transmission settings									
Eisele et al 2015; Eisele et al 2016	Zambia<br>Subnational: Lake Kariba in Southern Province, Zambia<br>Low (inland) & high	Impact Evaluation	2 rounds of MDA of and focal MDA (fMDA) with 3 day course of DHAP given as DOT to eligible population[12]	To quantify the relative effectiveness of MDA and fMDA against no mass treatment (standard of care) for reducing Plasmodium falciparum prevalence and incidence in low	Cluster randomized controlled trial of 60 health facility catchment areas (HFCAs), with a cross-sectional post intervention survey (part of parasite prevalence surveys) used to assess coverage among	Complete enumeration and geo-referencing of all households in the study area + Allocation of 10 HFCAs per transmission stratum in MDA, fMDA and control groups + Simple random	2,820 households	Post-MDA household survey (*based on MIS standardized survey items)*;<br>MDA campaign	• Household coverage by round<br>• Proportion of individuals aged ≥3 months, and households visited, offered the MDA and fMDA interventions within the target areas (Operational program coverage).<br>• Proportion of individuals (≥3 months) that agreed to participate in the MDA and fMDA interventions among all

[10] Excluding infants under 6 months, malnourished children, women in their first trimester of pregnancy, persons with fever or feeling unwell, persons who received ASAQ within the last month, and patients taking Zidovudine, Efavirenz or co-trimoxazole.
[11] Excluded children under 6 months of age
[12] Excluding children under three months and women in their first trimester of pregnancy

Study	Study Setting	Study Context	Intervention Details	Study Purpose	Study Design	Sampling Methods	Sample	Coverage data source	Indicators
	(lakeside) transmission settings			and high transmission settings	sample of participants.	sample of 47 households within each of HFCAs		records & census enumeration data	individuals eligible to participate in the intervention in the target population (Effective program coverage).
Bousema 2016	Kenya<br><br>Subnational: Western Kenya; highland fringe setting with low and heterogeneous malaria transmission	Impact Evaluation	Focal MDA with AL[13] as part of package of targeted interventions for 'hot spot' transmission zones[14] (fMDA + IRS + ITNs)	To determine the impact of interventions (larviciding, LLINs, IRS and fMDA) targeted to "malaria hotspots" on malaria transmission both inside hotspots and in surrounding communities.	Cluster-randomized controlled trial of 10 serologically defined hotspots,	Enumeration and geo-referencing of all compounds in study area	432 intervention compounds	MDA campaign records (*assumed*) + (*household survey for IRS & ITN indicators)*	• Proportion of compounds that participated in the screening of sentinel populations by RDT that prompted treatment<br>• Proportion of compounds with 1 RDT-positive inhabitant that were therefore eligible for MDA<br>• Proportion of the members of these eligible compounds who completed six doses of treatment, of which the morning doses (dose 1, 3, and 5) were observed and the afternoon doses were given without supervision, but blisters were checked.<br>• Proportion of respondents reporting sleeping under an LLIN the night before the survey<br>• Proportion of sleeping spaces sprayed with IRS
Finn et al. 2019	Zambia<br><br>Subnational: Lake Kariba in Southern Province, Zambia<br><br>Low (inland) & high (lakeside)	Impact Evaluation	2 rounds of MDA of and focal MDA (fMDA) with 3 day course of DHAP given as DOT to eligible population[15]	To compare estimates of MDA epidemiological coverage and program reach using a post-campaign survey, capture-recapture and satellite-enumeration methods	Cluster randomized controlled trial of 60 health facility catchment areas (HFCAs), with a cross-sectional post intervention survey used to assess coverage among sample of participants.	Simple random sample of 47 households within each of HFCAs for cross-sectional survey. Satellite enumeration of all households in 40 intervention HFCAs.	50,364 households from satellite enumeration, 1649 households from mid-point survey. A range of 14,921 to 17,883	Mid-point survey, MDA program records, satellite enumeration.	• Proportion of structures identified by satellite enumeration in intervention area that were visited by MDA campaign teams (Program reach - satellite enumeration)<br>• Proportion of surveyed households visited by MDA teams (Program reach - survey)<br>• Proportion of survey respondent reporting receiving

[13] Artemether-lumefantrine
[14] Pregnant or infants below 6 months of age were excluded from fMDA, but not other interventions
[15] Excluding children under three months and women in their first trimester of pregnancy

Study	Study Setting	Study Context	Intervention Details	Study Purpose	Study Design	Sampling Methods	Sample	Coverage data source	Indicators
	transmission settings				Capture-recapture method combining MDA program records and cross-sectional survey data.		households visited during each MDA round.		MDA drugs, among all residents surveyed. (Surveyed epidemiologic coverage)<br>• Proportion of total estimated population (number estimated from MDA campaign data plus imputed population not reached) who received MDA drugs
Intermittent preventive treatment in pregnancy (IPTp)									
Orobaton 2016	Nigeria<br>Subnational: Sokoto State	Pilot Evaluation	Community distribution through door to door DOT of 3+ doses of SP	To examine feasibility and acceptability of community-based SP delivery, and document associations with improved outcomes	Community and facility data on IPTp-SP distribution were collected in four local government areas (LGA) (3 intervention, 1 counterfactual) during 2015	Eligible LGAs purposively selected (high malaria in pregnancy prevalence & each represented a different senatorial zone)	25,572 pregnant women in the intervention LGAs and an estimated 5921 in the counterfactua l LGA were eligible to receive SP1	Program records (CHBV's standard outcome form)	• Proportion of pregnant women who receive ≥1 round IPTp<br>• Proportion of pregnant women who receive ≥3 round IPTp
Salomao 2017	Mozambique<br>Subnational	Routine Implement ation	ITNs & Clinic-based administrati on of at least 2 doses of SP via DOTS	To assess the uptake of IPTp and ITNs in pregnant women attending ANC services and correlate with ANC attendance and frequency of stock-outs in 22 health facilities in Mozambique.	Retrospective facility based cross-sectional study using program monitoring data conducted in 22 health units between July and December 2011.	Within each district, two health facilities were selected (one rural, one urban) which in total comprised 22 health facilities in 11 districts situated in 11 provinces. At each setting (rural or urban/sub urban), the health facilities with highest demand for ANC services were selected.	23,524 pregnant women attended first visit during study period	ANC logbooks; monthly district statistics and reports	• Proportion of pregnant women who receive 1 round IPTp<br>• Proportion of pregnant women who receive 2 round IPTp<br>• Proportion of ANC visits in which IPTp provided<br>• Proportion of pregnant women who received ITN at ANC visit
Indoor Residual Spraying									
Bridges 2018	Zambia<br>Subnational: Nchelenge and Samfya	Pilot Study	IRS:<br>Satellite enumeration + different	To assess the accuracy of the satellite-based enumeration in terms of its ability	Cross sectional household survey conducted 4 months post-IRS implementation used	Enumeration of all spray areas in each district + Two stage sampling process used to select spray	3,692 sprayable structures (ground enumeration);	Househol d survey + IRS card	• Proportion of surveyed households sprayed with IRS in in past four months [assumed, not specified]

Study	Study Setting	Study Context	Intervention Details	Study Purpose	Study Design	Sampling Methods	Sample	Coverage data source	Indicators
	districts in Luapela Province and Kasama district in Northern Province. Above average national malaria prevalence		levels of spatial aids: (1) tablet-based maps from satellite enumeration; (2) paper maps based on satellite enumeration; and (3) paper maps not based on satellite enumeration. Insecticide not specified	to identify sprayable structures; and measure the impact of varying degrees of spatial aids on community-level IRS coverage during the 2015 spray campaign in Zambia	to assess IRS coverage. Within selected spray areas, households enumerated during survey were compared to satellite enumeration for accuracy in identifying number of sprayable structures within a spray area	areas for data collection sample	3449 sprayable structures (satellite enumeration). 2679 households interviewed		• Sensitivity of satellite enumeration
Mumbengegwi 2018	Namibia<br><br>Subnational: Western Zambezi region of Namibia	Routine Implementation	IRS:<br>DDT (75%) or Deltamethrin	To determine (1) IRS coverage (administrative and reported), (2) its effects on malaria incidence, and (3) reasons for non-uptake of IRS in 2014-2015 malaria season	Cross-sectional household survey in four selected constituencies + administrative IRS coverage data based on MoHSS IRS records for the same areas.	A household census was conducted from Oct 2014-May 2015 (using the 2011 census data as a guide) with all consenting households eligible for participation in household survey		IRS Program records + Household survey	• Proportion of surveyed households sprayed with IRS in previous 12 months<br>• Proportion of sprayable structures sprayed (administrative)
Wagman 2018	Mali<br><br>Subnational: Segou Region in Central Mali	Pilot Study	IRS:<br>Bendiocarb in 2012 and 2013 and pirimiphos-methyl in 2014 and 2015	To assess the impact of IRS campaigns on malaria incidence rates reported through local and district health systems before and after spraying	Retrospective time series analyses using data from district routine health systems from January 2012-January 2016, with IRS coverage assessed using administrative data	Targeted district enumeration	N/A	IRS Program records	• Proportion of targeted houses sprayed; Reader referred to PMI reports for each year of IRS implementation
Nwe 2017	Myanmar	Routine Implementation	IRS + ITN: DDT (75%) and Fendona	To evaluate achievements and challenges of implementing	Descriptive study using annual records of National Malaria Control Programme	All program areas	52 study townships populated by	IRS Program records + ITN	• Households/Buildings sprayed, annually

Study	Study Setting	Study Context	Intervention Details	Study Purpose	Study Design	Sampling Methods	Sample	Coverage data source	Indicators
	Subnational: 52 townships of artemisinin-resistant "containment areas"			measures to prevent and control spread of artemisinin resistance in Myanmar.	(NMCP) data for 2010-2014 from artemisinin-resistant containment areas		8.7 million inhabitants	distribution (NMCP data)	• Proportion of at risk population covered by distributed LLINs
Sande 2017	Zimbabwe<br><br>National	Routine Implementation	IRS + ITN: pyrethroid insecticides	To analyze changes in malaria morbidity trends in Zimbabwe based on surveillance data and reorient strategies for malaria elimination.	Document review using data from the National Malaria Control Programme (NMCP) annual reports and MICS 2008, 2012, 2014	All program areas/No sampling	Not specified	IRS Program records	• Proportion of targeted rooms sprayed (administrative)<br>• Proportion of population protected by IRS (administrative)<br>• HH ownership of ≥1 ITN/LLIN<br>• Individual using ITN/LLIN on previous night
Raman 2016	South Africa<br><br>Subnational	Routine Implementation	IRS:<br><br>Pyrethroids and DDT and, in certain instances carbamates	To assess the progress made towards elimination, and suggest future strategies	Desk Review of national malaria case data and annual spray coverage data from 2010 until 2014	All program areas/No sampling	Not specified	IRS Program records	• Proportion of sprayable structures sprayed (administrative)
Mangam 2016	Mali<br><br>Subnational: Koulikoro district	Pilot Study	IRS:<br><br>Pilot implementation of voice and/or text messages received on cell phones vs. door-to-door mobilization for an indoor residual spraying (IRS) campaign	To evaluate the use of mHealth tools for IRS mobilization in Mali.	Descriptive study comparing structure preparedness and coverage in the pilot intervention villages compared with villages mobilized through standard door-to door mobilization methods.	Six villages selected purposively and all households within enumerated.	576 structures in 3 intervention villages; 877 structures in 3 control villages	IRS Program records	• Proportion of sprayable structures sprayed (administrative)

Study	Study Setting	Study Context	Intervention Details	Study Purpose	Study Design	Sampling Methods	Sample	Coverage data source	Indicators
Johns 2016	Ethiopia<br><br>Subnational: Oromia region	Pilot Study	IRS:<br><br>Pilot of IRS as part of government-funded community-based rural health services program	To test the effectiveness and efficiency of integrating IRS into the government-funded community-based rural health services program.	Between 2012 and 2014, PMI conducted a mixed-methods study in 11 districts of Oromia region to compare district-based IRS and community-based IRS models.	Six villages were purposively selected for the intervention with five matched villages receiving traditionally implemented IRS.	N/A	IRS Program records	• Proportion of eligible structures sprayed (administrative)<br>• Proportion of population protected by IRS (administrative)
Katureebe 2016	Uganda<br><br>Subnational: 3 sub-counties in Uganda, Varying transmission intensities	Routine Implement ation	IRS + ITN	To monitor for changes in the burden of malaria following the scale-up of LLIN distribution and IRS	Comprehensive malaria surveillance conducted at three sites from October 2011 to March 2016; repeated cross-sectional community surveys in 2012, 2013, and 2015 used to estimate the coverage level of key malaria control interventions + cohort of children 0.5–10 y enrolled August to September 2011.	For cross sectional surveys, households randomly selected from study enumeration list and sequentially screened until 200 households enrolled. For cohort, Children 0.5–10 y were enrolled from 100 households from each study site	200 households/ survey/year	Househol d survey + Program records + Cohort assessme nt	• Proportion of surveyed households sprayed with IRS in last 12 months<br>• Proportion of eligible structures sprayed (administrative)<br>• HH ownership if ≥1 ITN/LLIN<br>• HH with universal coverage of ITNs/LLINs (≥1 net for every 2 people in HH)<br>• Individual using ITN/LLIN on previous night
**ITN/LLIN – Primary data used for coverage estimates**									
Aregawi 2017	Ghana<br><br>Subnational: Sample hospitals representing the three epidemiologi cal zones in Ghana	Routine Implement ation	IRS & ITNs:<br><br>NMCP intervention s (ACT, LLINs, IRS).	To assess the impact of ACT, LLINs and IRS on malaria cases, admissions and deaths using data from district hospitals.	Analysis of records of malaria cases and deaths and availability of ACT in 88 hospitals, as well as district level records for ITN distribution and IRS.	Not specified, but hospitals only represent a sample	88 hospitals	District records	• Proportion of population of all ages potentially protected by LLINs, assuming each LLIN covered 1.8 persons and lasted 3 years<br>• (IRS also collected but not fully described in this paper)

Study	Study Setting	Study Context	Intervention Details	Study Purpose	Study Design	Sampling Methods	Sample	Coverage data source	Indicators
Delil et al 2016	Ethiopia<br><br>Subnational	Routine Implement ation	ITN/LLIN	To assess magnitude and factors affecting malaria in low transmission areas among febrile cases attending public health facilities in Hadiya Zone, Ethiopia	Facility based cross-sectional study was conducted in Hadiya Zone from May 15 to June 15, 2014	Health facilities were selected using simple random sampling, with systematic random sampling used to select febrile patients attending in selected health centers	422	Facility survey	• HH ownership of ≥1 net, including ITN/LLIN<br>• Individual using ITN/LLIN on previous night (father, mother, <5, ≥5, other)
Gama et al 2016	Kenya<br><br>Subnational	Process/Im pact	ITN/LLIN	To find the most cost-effective mix of LLIN distribution channels that would maximize coverage for beneficiaries, estimating distribution cost and the effectiveness of different LLIN distribution models, and comparing them in an economic evaluation.	Retrospective economic evaluation of the LLIN distribution channels in Busia County, western Kenya. Coverage outcomes triangulated from a nationally representative cross-sectional survey, a cross-sectional survey administered to a subsample of beneficiaries in Busia County, and distributors' records.	Nationally representative survey: Two stage cluster sampling (assumed); Busia survey: Randomly selected households in Busia County	Nationally representativ e survey: 6300 households; Busia survey: 592 households	Househol d survey *(DHS/MIS)* +<br><br>Househol d survey + distributo rs records	• HH ownership of ≥1 ITN/LLIN<br>• HH with universal coverage of ITNs/LLINs (≥1 net for every 2 people in HH)<br>• Individual using ITN/LLIN on previous night
Kamau et al 2017	Kenya<br>Subnational	Routine Implement ation	ITN/LLIN	To evaluate trends in bednet ownership and usage, and the effect of bednets on the incidence of malaria hospitalization in children < 5 years within the Kilifi Health and Demographic	Eight routine enumeration rounds of the Kilifi Health and Demographic Surveillance System (KHDSS) between 2008 and 2015 and four serosurveys among randomly selected children aged 0-15 in the KHDSS register.	Population of the Kilifi Health and Demographic Surveillance Site	KDHSS population =260,000 residents; 1986 children 0–15 years of age in sero-surveys	Routine surveillan ce data	• Individual using ITN/LLIN on previous night<br>• Other: Proportion of respondents with bednet available for use in an individual's usual sleeping area

Study	Study Setting	Study Context	Intervention Details	Study Purpose	Study Design	Sampling Methods	Sample	Coverage data source	Indicators
				Surveillance System (KHDSS).					
Lalji et al 2016	Tanzania<br>Subnational	Pilot Study	ITN/LLIN	To evaluate a school-based approach to ITN distribution in the country's Southern Zone starting in 2013, called the School Net Program 1 (SNP1)	Case study describing intervention roll-out with monitoring data used to calculate coverage estimates.	All schools included in the intervention (census)	n/a	Program/campaign records	• Other: Proportion of eligible students and teachers who received LLINs
Machini et al 2016	Kenya<br>National	Routine Implementation	ITN/LLIN	To analyze from 2011-2015, the trends in number and proportions of reported outpatient malaria cases (confirmed and clinical) stratified by age group and compared to mass LLIN coverage in the different malaria epidemiological zones of Kenya	Retrospective study using routinely reported national program data.	All individuals with data in DHIS2 for outcome data	n/a	NMP activity reports	• Proportion of population potentially protected by LLINs
Mangeni et al 2016	Kenya<br>Subnational	Pilot Study	ITN/LLIN	To describe the development, testing and validation of a rapid assessment tool to collect actionable information at local levels for a quick evaluation of potential barriers to malaria prevention	Community health volunteers recruited patients from health facilities and subsequently followed them up to their homes to conduct a rapid assessment. Sampling and analysis of the results of the survey were based on Lot Quality Assurance	Individuals with laboratory-confirmed malaria in six peripheral health facilities located within six sub locations recruited	268 included	Rapid Assessment tool	• Other: Proportion of sleeping spaces with nets<br>• Other: Proportion of people inn households who did not sleep under a net last night

Study	Study Setting	Study Context	Intervention Details	Study Purpose	Study Design	Sampling Methods	Sample	Coverage data source	Indicators
Okoyo et al 2015	Kenya<br><br>Subnational	Process/Impact evaluation	ITN/LLIN	Describe malaria among Kenyan schoolchildren living in areas of intense malaria transmission, and their reported use of ITNs.	School based cross sectional survey (EAG)	Randomly selected schools from each county, then 10 boys and 10 girls randomly selected from classes 2 to 6 to provide circa 100 children/school. In small schools, all the students in classes 2–6 were recruited.	5188 children from 54 schools	School based Survey	• HH ownership of ≥1 ITN/LLIN<br>• Universal coverage of ITN/LLIN<br>• Individual using ITN/LLIN on previous night
Peeters Grieten 2015	Cambodia<br><br>Subnational	Routine Implementation	ITN/LLIN	To characterize the different mobile groups and their vulnerability to malaria in the context of persisting residual transmission foci	Mixed method design using ethnographic interviews and cross-sectional surveys of migrant groups (Khmer) and indigenous groups traditionally resident in the study area.	**Khmer Migrant Survey:** In each study site, all Khmer working households (HH) that could be located on the plantation were invited to participate in the survey.<br><br>**Indigenous population survey**: A random sample of 900 individuals from different households were selected from the population census of the 113 villages included in the MalaResT study in 2012 and invited to participate in the survey.	186 Khmer household leaders in 67 plantations; 824 individuals in Indigenous population survey	Questionnaire<br><br>Household survey	• Individual using ITN/LLIN on previous night<br>• Other: Received bed net from VMW program<br>• Other: Forest farm HH ownership of bed nets (for sleeping huts in deep forest agricultural plots used for sleeping during harvest seasons)

Table S2. Intervention Coverage Measurements of Included Studies

Indicators	Numerator	Denominator	Indicator data source	Challenges to coverage estimation	Study/ies
SMC: Children receiving first SMC dose over all SMC rounds for transmission season (3 or 4 rounds)					
Proportion of eligible children who received SMC during all three monthly rounds	Number of courses of SMC administered (captured on register and on the mother's card)	Number of SMC-eligible children in DSS	Administrative Records (registers) and DSS population estimates	Adequately Defined, though— Authors note that DSS denominator estimates were based on the total population usually resident in the study area, which may have overestimated the population at risk, given mobility during the transmission season, and consequently underestimated coverage.	Ba 2018
SMC: Children receiving first SMC dose in specific SMC round					
Proportion of children who received the first dose of each treatment cycle during the transmission season, calculated per treatment cycle	Number of children aged 3-59 months who received the first dose of AQ+SP	Expected number of children aged 3-59 months in the locality during the current transmission season	Administrative Records (daily data transmitted during SMC campaign cycles) and adjusted census data	Not Adequately Defined (monthly estimates exceeded 100%) – No census was undertaken as part of routine SMC procedures in 2014 or 2015 and so the estimate of the target population relied on adjusting the last population census (*adjustments not described.*) Further, age-eligibility criteria were not always followed leading to treatment of off-target children.	Compaore 2017
MDA: People receiving MDA drug during specific treatment round					
Coverage of MDA in each round	Number of full ASAQ treatment courses distributed	Number of people targeted for MDA	MDA campaign records (NMCP) + Target population was determined based on the Household Registration used for 2014 national LLINs mass campaign	Some— The authors noted that cross-over from non-MDA districts and out-of-date census data may have impacted coverage estimates	Aregawi 2016
Proportion of population that received each round of MDA out of total population	The number of target population treated at each of three rounds	Total population, including those not eligible for MDA	MDA Campaign records + pre-MDA census records (2012 census data compiled by malaria control teams in the Union of Comoros)	None	Deng 2018
Proportion of study participants with at least one complete round (two or more rounds; or all three rounds) of MDA treatment out of all study participants	Number of respondents who participated in at least one round of MDA	Eligible village population present at any point during MDA intervention period	MDA campaign records	None, though— Coverage estimates exclude those ineligible for MDA, including those away at the time of the intervention	Tripura 2018
Proportion of respondents who participated in at least	Number of respondents who participated in at	Eligible village population	MDA campaign records	None, though— Coverage estimates exclude those ineligible for MDA	Adhikari 2017

Indicators	Numerator	Denominator	Indicator data source	Challenges to coverage estimation	Study/ies
one round of MDA and blood survey out of all eligible participants	least one round of MDA and blood survey				
Proportion of respondents who participated in all three rounds of MDA and blood survey out of all eligible participants	Number of respondents who participated in all three rounds of MDA and blood survey	Eligible village population	MDA campaign records	None, though—<br>Coverage estimates exclude those ineligible for MDA	Adhikari 2017
Proportion of targeted population that received treatment out of all people enumerated in four districts during MDA	Number of targeted population that received first day of MDA (DOT) and doses for day 2-3.	Total population, including those not eligible for MDA	MDA campaign records	Some—<br>Population (census) estimates were presented with confidence intervals	Ali 2007
Coverage of MDA under DOT	Number of people who took the first dose of the medicine under DOT on the day of visit by the drug distributors	Number of people who received full treatment courses	MDA campaign records (NMCP)	None, though—<br>Only captured during second MDA round	Aregawi 2016
Operational program coverage (proportion of eligible population reached with intervention)	Number of individuals aged three-months-old and older, and households visited, offered the MDA and fMDA interventions	Number of eligible individuals aged three-months-old and older, and households within the target areas	MDA & fMDA campaign records + census enumeration data	No coverage estimate provided (protocol)	Eisele 2015
Effective program coverage (proportion of eligible population that participated in intervention)	Number of individuals (aged three-months and older) that agreed to participate in the MDA and fMDA interventions	All individuals eligible to participate in the intervention in the target population.	Household enumeration for the sampling frame	No coverage estimate provided (protocol)	Eisele 2015
Adjusted epidemiological coverage	Number of individuals (aged three-months and older) that agreed to participate in the MDA and fMDA interventions	All individuals reached by MDA teams plus estimated population not reached by MDA estimated from capture-recapture method	MDA campaign records and capture-recapture estimates	Capture-recapture requires matching of individual-level data (in this case, individuals registered for MDA and participants in separate cross-sectional survey)	Finn 2019
Program reach (using capture-recapture)	Number of households reached by community health workers during MDA campaign	Number of households estimated to exist using capture-recapture methods	MDA campaign records and capture-recapture estimates	Capture-recapture requires matching of individual-level data (in this case, individuals registered for MDA and participants in separate cross-sectional survey)	Finn 2019
MDA: People completing full course of treatment					

Indicators	Numerator	Denominator	Indicator data source	Challenges to coverage estimation	Study/ies
Proportion of respondents that completed three consecutive doses of anti-malarials in any one round (and all three rounds) out of total respondents	Number of heads of household that completed daily MDA Dose (DOT) over three days (and three rounds)	Number of heads of household in the MDA database	MDA campaign records	Some – Coverage estimates restricted to heads of households with interview data and an entry in the study database (10/388 respondents did not have campaign record data). No mention of how households were enumerated, whether all households were able to be interviewed, or whether ineligible respondents were included in the denominator.	Kajeechiwa 2016
Focal MDA					
Proportion of compounds that participated in the screening of sentinel populations	Number of compounds that participated in the RDT screening	Number of compounds in study zones	MDA campaign records	None	Bousema 2016
Proportion of compounds with 1 RDT-positive inhabitant that were therefore eligible for MDA	Number of compounds eligible for fMDA (≥1 RDT-positive inhabitant)	Number of compounds that participated in the RDT screening	MDA campaign records	None	Bousema 2016
Proportion of members of eligible compounds who completed full course of treatment	Number of household members that completed full course of antimalarials (six doses of treatment, of which the morning doses (dose 1, 3, and 5) were observed and the afternoon doses were given without supervision but blisters were checked.)	Number of members in compounds eligible for fMDA (≥1 RDT-positive inhabitant)	MDA campaign records	None	Bousema 2016
IPTp: Pregnant women receiving ≥1, ≥2, ≥3 rounds IPTp					
• Proportion of pregnant women who receive ≥1 round IPTp<br>• Proportion of pregnant women who receive ≥3 rounds IPTp<br>(Method 1 – household registration system database)	Number of pregnant women receiving specified number of IPTp doses	Total number of surveyed women eligible to receive SP (clear cut history of quickening without history of SP administration within last 4 weeks or history of reaction to sulphur-containing drugs)	Program outcome form + HRS database<br>Households were mapped and all women of reproductive age including those eligible for IPTp, were enumerated and registered in a household registration system database.	Some—<br>*In one of the three intervention areas, there were a number of households whose pregnant women were served with SP and not captured in the intervention tracking database due to delays in enumeration. Consequently, these were not included in the denominator, and quite likely yielded an overestimated SP1 coverage.*	Orobaton 2016
• Proportion of pregnant women who receive ≥1 round IPTp	Number of pregnant women receiving	The estimated number of pregnant women eligible to receive SP1	Program outcome form + modified population estimates	Some—<br>*The house-to-house enumeration, in two of the three intervention areas, showed a higher number of eligible*	Orobaton 2016

Indicators	Numerator	Denominator	Indicator data source	Challenges to coverage estimation	Study/ies
• Proportion of pregnant women who receive ≥3 rounds IPTp (Method 2 – Census projections)	specified number of IPTp doses	over the life of the project (clear cut history of quickening without history of SP administration within last 4 weeks or history of reaction to sulphur-containing drugs)	Official population estimates (Sokoto State Government) were multiplied by 5% (assumed proportion of population that is pregnant) and 94% (proportion of pregnant women eligible for the intervention) and prorated by the 8 months of the project.	*women than were estimated from official population estimates, suggesting that there may have been an under estimate of the number of eligible women in the counterfactual area. If this was the case, coverage rates of IPTp in the counterfactual area would be even lower than estimated.*	
• Proportion of pregnant women who receive ≥1 round IPTp<br>• Proportion of pregnant women who receive 2 rounds IPTp<br>(Method 3 – ANC records)	Number of pregnant women receiving specified number of IPTp doses	Number of pregnant women attending ANC service for their 1st visit	Antenatal logbooks + monthly district reports	Not adequately defined—<br>The denominator was defined as 'pregnant women attending first ANC visit' and used to assess coverage for receipt of first and second dose of IPTp; There was no mention of whether the denominator was limited to those eligible for IPTp (e.g. within their first trimester, co-trimoxazole use). The same denominator was used for ITN and IPTp coverage, suggesting it is not limited to those eligible for IPTp, but all presenting pregnant women.	Salomao 2017
IPTp: Other indicators					
Proportion of ANC visits in which SP was provided	Number of ANC visits in which second dose of IPTp was provided	Number of ANC visits conducted and recorded	Antenatal logbooks + monthly district reports	See previous comment re Salomao	Salomao 2017
IRS: Proportion of structures sprayed (per spray cycle)					
Proportion of structures sprayed in a given area (village, etc)	Total number of structures sprayed	Total # of eligible structures targeted (enumerated by Spray Operators)	Program records – (implementing partner or NMCP)[16]	Some—<br>• Wagman 2018 references campaign records as the IRS coverage source. The underlying report indicates that enumeration was not conducted in 2012, so the denominator for the spray coverage rate was the number of structures found during the 2011 spray campaign<br>• Mangam 2016 noted that in the district with the lowest coverage: *Spray Operators encountered numerous structures where the owners/head of household were in their fields working and would not return home until after spray teams had left the area.*	Wagman 2018; Mangam 2016; Johns 2016; Katureebe 2016; Raman 2016; Sande 2017; Mumbengegwi 2018
Proportion of structures sprayed annually	Total number of covered	Total number of covered households/buildings (including refused)	Program records (NMCP annual records)	Some—	Nwe 2017

[16] Wagman, Mangam & Johns relied on PMI AIRS campaign records, while Katureebe, Sande and Raman relied on National Malaria Control Program (NMCP) data, and Mumbengegwi relied on IRS coverage routinely reported by the health districts to the Ministry of Health and Social Services

Indicators	Numerator	Denominator	Indicator data source	Challenges to coverage estimation	Study/ies
	households/buildings sprayed			• No true coverage estimate presented as the denominator was not defined for the IRS campaign<br>• Study relied on data from NMCP and the authors noted: some data from other implementation partners were not available to them.<br>• The authors noted that mobile populations were not accounted for in these estimates as the information was not readily available.	
Proportion of clusters sprayed by IRS	Total number of structures sprayed	Total number of sprayable structures	Study records	*IRS coverage estimates not clearly defined, but measured as part of a randomized controlled trial.*	Pinder
**IRS: Proportion of targeted population protected by IRS**					
Proportion of targeted population protected by IRS per spray cycle	Number of people protected	Total number of people in target population	NMCP annual reports, unpublished	None	Sande 2017
Proportion of targeted population protected by IRS per year	Number of people protected	Total number of people in target population	Implementing partner campaign records	None	Johns 2016
**IRS: Other Measures**					
Measure of sensitivity of satellite enumeration	Total number of houses enumerated by satellite	Total number of houses in the target area	Satellite enumeration records + ground enumeration records	The number of houses enumerated by satellite but not found on the ground is reported as false negative	Bridges 2018
**ITN: HH ownership of ≥1 ITN/LLIN**					
Modeled ITN coverage (Percent of children living in a household covered by an ITN according to modeled estimates combining household ITN ownership and ITN distribution data adjusted for population and a lag factor (low/high))			Household survey data (DHS/MICS) + administrative records of number of ITNs distributed	*Data on annual, district-level ITN distribution were used and a decay factor was applied as per previously published methodology. This decay factor accounts for some loss of ITNs per year (either due to attrition or physical deterioration) and includes ITNs distributed in previous years to estimate total annual ITN ownership. ITN distribution data were also adjusted for mid-year district-level population available through census data. Finally, the values were included with ITN ownership data from the 2010 DHS and the 2006 MICS in Loess regression models to create best-fit district-year estimates of ITN ownership.*	Florey 2017
ITN: Individual using ITN/LLIN on previous night					
Proportion of population that slept under an ITN the previous night (Method 4: unspecified indicator source + routine surveillance data)	Number of individuals who slept under an ITN the previous night	Total number of individuals who spent the previous night in surveyed households	Routine surveillance data (KHDSS) using household survey	Part of Kilifi Health and Demographic Surveillance System (KHDSS) One resident was allowed to respond for all other residents of the same homestead	Kamau et al 2017

Indicators	Numerator	Denominator	Indicator data source	Challenges to coverage estimation	Study/ies
ITN: Other Indicators					
Proportion of population of all ages potentially protected by LLINs assuming each LLIN covered 1.8 persons and lasted 3 years	Number of LLINs distributed in district	Prospective district population was derived from the 2010 Ghanaian census, using the United Nations growth rate for Ghana	District records on ITN distribution	None	Aregawi 2017
Proportion of eligible students and teachers who received LLINs in school bed net distribution campaign	Number of students and teachers with recorded receipt of net	Total number of students and teachers in targeted schools	Program/campaign records	None	Lalji et al 2016
The proportion of population potentially protected by LLINs in a given year per region	Number of LLINS distributed per year per region multiplied by 1.8	Population data per region (not specified)	Routine surveillance data (DHIS2)	The proportion of people potentially receiving LLINs in a given year per region was calculated by assuming that each LLIN distributed protected 1.8 persons for three years	Machini et al 2016
